# Modeling Tau-Mediated Pathology in Monkey Brain Slices

**DOI:** 10.3390/biology15171496

**Published:** 2026-09-02

**Authors:** Mingtian Pan, Qintian Guo, Peisi Huang, Xiang Han, Sitong Yang, Fengwei Sun, Jiawei Zhang, Laiqiang Chen, Bang Li, Dajian He, Shihua Li, Xiao-Jiang Li, Xiangyu Guo

**Affiliations:** 1 Ministry of Education CNS Regeneration Collaborative Joint Laboratory, Guangdong-Hongkong-Macau Institute of CNS Regeneration, Jinan University, Guangzhou 510632, China; panmingtian@outlook.com (M.P.); guoqt@stu.jnu.edu.cn (Q.G.); hps0629@stu2024.jnu.edu.cn (P.H.); hanyh@stu2024.jnu.edu.cn (X.H.); yangsitong@stu2023.jnu.edu.cn (S.Y.); fengweisun@jnu.edu.cn (F.S.); aslanzz@163.com (J.Z.); kagami233@stu2019.jnu.edu.cn (L.C.); libang2012@sina.com (B.L.); dajian.he@lglab.ac.cn (D.H.); lishihualis@jnu.edu.cn (S.L.); 2 Guangdong Key Laboratory of Non-Human Primate Research, Guangdong-Hongkong-Macau Institute of CNS Regeneration, Jinan University, Guangzhou 510632, China; 3 Lingang Laboratory, Shanghai 200031, China

**Keywords:** non-human primate, neurodegenerative diseases, brain slice culture, in vitro model, tau phosphorylation

## Abstract

Tau pathology is a hallmark feature of Alzheimer’s disease (AD) and is tightly associated with clinical manifestations. No disease-modifying therapy is currently available for AD, partly due to prominent interspecies differences in tau expression patterns between rodents and primates. To facilitate the investigation of primate-specific tau pathology, we developed an in vitro platform using monkey (Macaca fascicularis) brain slice cultures (BSC) that preserve native three-dimensional tissue architecture and maintain functional networks closely mirroring in vivo conditions. We systematically evaluated various adeno-associated virus (AAV) serotypes and promoter combinations to achieve efficient transgenic tau expression. Our data confirm that the monkey BSC system is highly amenable to AAV-mediated gene delivery, exhibiting robust transduction of glial cells with minimal cytotoxicity and allowing for the examination of glial pathology induced by disease-associated proteins. Notably, this platform is applicable to both juvenile and aged specimens, enabling comparative analyses of age-dependent susceptibility to pathological insults. Following overexpression of mutant human tau, we observed time-dependent tau pathology development within 2–4 weeks post-infection, including hyperphosphorylation and aggregation. This in vitro primate model offers a unique opportunity to dissect primate-specific pathological events. Furthermore, the scalability and reproducibility of this system render it ideally suited for drug screening, providing a more translationally relevant preclinical bridge between rodent models and human clinical trials.

## Simple Summary

Early-stage tau-related pathogenesis research remains challenging, largely due to the scarcity of appropriate animal models and the extremely limited accessibility of clinical specimens. Here, we established a nonhuman primate brain slice culture model that recapitulates early tauopathy with minimal animal usage and a short experimental timeline. This model can serve as a pivotal platform bridging rodent and human tau research, facilitating translational studies as well as elucidating primate-specific molecular mechanisms underlying tauopathy.

## 1. Introduction

Alzheimer’s disease (AD) predominantly manifests after the age of 65, and no current treatment can postpone, halt, or reverse its pathological progression [1,2]. Aging is the single greatest risk factor for AD. As the global population grows and healthcare advances, the elderly population is expanding rapidly; it is projected that over 153 million people will suffer from AD in the coming decades, placing an immense burden on healthcare systems and individual families, both economically and emotionally [3,4,5]. Despite substantial efforts to develop effective therapies, little success has been achieved to date [6]. A major contributing factor is the incomplete understanding of the underlying molecular etiology. Extracellular amyloid-β plaques and intracellular neurofibrillary tangles are the two pathological hallmarks of AD, and mounting evidence indicates that tau pathology is more closely associated with disease progression [7]. However, tau expression patterns differ significantly between adult primates and rodents: macaque monkeys express 3R and 4R tau at an approximate 1:1 ratio, whereas rodents express only 4R tau [8], underscoring the unique relevance of primate models for studying tau pathology in AD.

Although neuronal dysfunction and degeneration are central concerns in AD, glial cells play a cardinal role in the initiation and progression of pathology [9,10,11,12,13,14]. The morphology, quantity, and distribution of glial cells in the primate central nervous system differ significantly from those in rodents [15,16,17], underscoring the unique value of non-human primate (NHP) models in complementing our understanding of AD pathogenesis. NHPs are among the most relevant animal models for biomedical research, owing to their evolutionary proximity to humans, as well as their emotional and behavioral complexity, which are underpinned by sophisticated cerebral structure and functional integration [18,19,20,21,22]. Recently, with advances in gene editing and NHP-assisted reproductive technologies, several transgenic and gene-knockout monkey models of neurological diseases have been generated [23,24,25,26,27,28,29,30,31,32,33,34,35], providing novel and in-depth mechanistic insights. Although numerous rodent AD models have contributed to our understanding of pathological mechanisms, they can hardly recapitulate the typical neuronal loss observed in human AD [36,37,38,39]. Moreover, while no rodent naturally develops AD-like pathology at the end of its lifespan, a subset of aged NHPs does display hallmark pathological features, implying that NHPs possess key endogenous contexts permissive for AD pathogenesis [19,40,41,42,43,44,45,46,47,48,49].

Although transgenic and gene-edited NHP models of AD are highly valuable, it is very difficult to obtain large cohorts of such models, as gestation, generation time, and lifespan in NHPs are considerably longer than those in rodents, litter sizes are much smaller, and animal resources are limited and costly [50]. Neuron–glia co-culture systems or brain organoids can serve to study mutual interactions [51,52]; however, these models do not fully recapitulate the original cellular contact patterns, and cell morphology and physiological states also differ from the in vivo condition. Brain slice culture is an intermediate in vitro model that preserves original cell localization and neuron–glia interactions [53,54,55]. This technique has been utilized in neuroscience research for decades [53,54,55], but predominantly on neonatal rodent specimens for electrophysiological studies. Recently, mouse brain slices have been shown to be applicable for investigating neurodegeneration [56,57,58,59,60,61,62]. However, to the best of our knowledge, cultured monkey brain slices have not yet been reported for studying neurodegenerative processes. In this study, we developed an NHP brain slice culture system and compared different adeno-associated virus (AAV) serotypes and promoters to optimize gene delivery efficiency. We demonstrated that glial cells can be effectively transduced by AAV and that age-dependent tau pathology can be recapitulated in this system. This platform offers an alternative approach to investigate primate-specific pathology induced by disease-associated proteins and to develop therapeutic strategies.

## 2. Materials and Methods

### 2.1. Monkey Tissues

All animals were housed under standard conditions in Guangdong Landau Biotechnology Co., Ltd. (Guangzhou, China), an Association for Assessment and Accreditation of Laboratory Animal Care International (AAALAC) fully accredited breeding company. Landau Company houses over 3000 cynomolgus macaques. As macaques are group-living animals with strict social hierarchies, aggressive rank-related fighting occasionally occurs, resulting in severe physical injuries in some individuals. A few neonatal and aged animals may also succumb to spontaneous illness. All tissue specimens were collected post-euthanasia, which was performed by veterinarians following humane endpoints. Cerebral tissues were promptly isolated from accidentally injured or heavily ill cynomolgus monkeys after euthanasia and transported to the laboratory at 4 °C. A total of nine animals across six distinct age groups were used in this study. All experimental protocols were reviewed and approved by the Institutional Animal Care and Use Committee of Landau Biotechnology Co. Ltd. (LDACU20201216-1) in advance. Studies were implemented in compliance with the National Institutes of Health (USA) Guide for the Care and Use of Laboratory Animals (8th edition, 2011), as well as the ARRIVE guidelines for reporting animal research.

### 2.2. Antibodies and Reagents

Antibodies used in this study are: mouse anti-Vinculin (MAB3574, MED millipore, Billerica, MA, USA), rabbit anti-glial fibrillary acidic protein (GFAP) (ab7260, Abcam, Cambridge, UK), rabbit anti-NeuN (ab177487, Abcam), rabbit anti-IBA1 (019-19741, Wako Chemicals, Osaka, Japan), rabbit anti-pMAP2 (ab96378, Abcam), mouse anti-tau(A-10) (sc-390476, Santa Cruz, Dallas, TX, USA), rabbit anti-pTau (AT8, MN1020, ThermoFisher, Waltham, MA, USA), rabbit anti-RFP (Rockland, 600-401-379, Limerick, PA, USA), and guinea pig anti-c-Fos (Synaptic Systems SY-SY, 226004, Göttingen, Germany). HRP-conjugated secondary antibodies are goat anti-mouse and goat anti-rabbit (Bosterbio, Wuhan, China). Fluorescence-conjugated secondary antibodies are donkey anti-mouse Alexa Fluor 488 or 594, donkey anti-mouse Alexa Fluor 488 or 594, and donkey anti-guinea pig Alexa Fluor488 or 594.

### 2.3. Western Blotting

Tissues were homogenized in RIPA buffer (50 mM Tris, pH 8.0, 150 mM NaCl, 1 mM EDTA pH 8.0, 1 mM EGTA pH 8.0, 0.1% SDS, 0.5% DOC, 50 mM NaF and 1% Triton X-100) containing Halt protease inhibitor cocktail (Thermo Scientific, Waltham, MA, USA) and protease inhibitor cocktail (78–430, Pierce, Rockford, IL, USA) and 1 mM phenylmethylsulfonyl fluoride (PMSF) (P-7626, Sigma, St Louis, MO, USA). The lysates were incubated on ice for 30 min, sonicated, and centrifuged at 12,000× *g* for 10 min. Equal amounts of protein were loaded on GenScript (Nanjing, China) Tris-glycine (4–12%) gels for SDS–polyacrylamide gel electrophoresis. Proteins transferred to nitrocellulose blots were incubated with primary antibodies in 3% bovine serum albumin/PBS overnight at 4 °C. Following incubation, the nitrocellulose blots were washed, and secondary HRP-conjugated antibodies (Bosterbio, Pleasanton, CA, USA) were added in 5% milk for 1 h. ECL-plus GE Healthcare (Little Chalfont, Buckinghamshire, UK) and the chemiluminescence imaging system (Clinx, Shanghai, China) were then used to reveal immunoreactive bands on the blots. All original WB images are provided at Supplementary Section.

### 2.4. Immunofluorescence and Immunohistochemistry

Tissue sections were washed 3 times with PBS, permeabilized and blocked with 0.3% Triton X-100 and 3% BSA for 2 h, and then incubated overnight at 4 °C with the primary antibody. For immunofluorescence, tissues were washed 3 times with PBS and incubated with corresponding fluorescent secondary antibodies for 2 h at room temperature. Nuclei were counterstained with DAPI (Beyotime, Shanghai, China).

Immunohistochemistry was performed using the VECTASTAIN ELITE ABC Universal Kit (Vector Laboratories, Peterborough, United Kingdom). Brain slices were incubated overnight at 4 °C with primary antibodies, followed by incubation with poly-HRP-conjugated IgG (anti-mouse or anti-rabbit, 1:1000, Dako, Santa Clara, CA, USA). DAB (3,3′-diaminobenzidine) staining was used for chemiluminescent detection. Images were obtained by an automated slide scanner (TissueFAXS Whole Slide Scanner; TissueGnostics, Vienna, Austria).

### 2.5. Electron Microscopy

Monkey BSCs with a tau transgene and wild type were collected at 10 days in vitro (DIV) and treated with 2.5% glutaraldehyde/0.1 mol/L PB overnight at 4 °C; then BSCs were sectioned into 50 μm using a vibratome (Leica, VT1000s, Ladenburg, Germany) and the sections were processed for electron microscopic examination. In brief, all sections were osmicated in 1% OsO4 in 0.1 mol/L PB and embedded in Eponate12 (Ted Pella). The dried brain sections were cut into ultrathin sections (60 nm) with a Leica Ultracut S ultramicrotome under a Hitachi H-7500 transmission electron microscope equipped with a Gatan Bio-Scan CCD camera (Tokyo, Japan).

### 2.6. Organotypic Monkey Brain Slice Culture and AAV Viral Infection

The brain cortex of a male or female adult monkey was carefully dissected on ice and directly transferred into ice-cold artificial cerebrospinal fluid (aCSF) (SL6630X-500mL, Coolaber, Beijing, China) equilibrated with carbogen (95% O_2._, 5% CO_2_). The tissues were cut at 300 μm thickness using a VT1200S vibrating blade microtome (Leica, Germany). From each animal, 8 or 10 coronal slices (all even numbers) were obtained. These slices were randomly and evenly divided into two groups: one half was designated as the control group (AAV-Ctrl) and the other half as the experimental group for tau overexpression (tgTau). Each individual slice was then transferred onto a separate 30 mm Millicell membrane insert (0.4 μm; Millipore, Bedford, MA, USA) placed in a well of a six-well plate with 1.5 mL medium (Neurobasal-A/DMEM 1:1, 5% fetal bovine serum, 15% heat-inactivated horse serum, 1% N2 supplement, 2% B27 Plus supplement, 2 mM L-GlutamaX, 5 ng/mL Recombinant Human GDNF (R&D Systems, 212-GD, Minneapolis, MN, USA), 5 ng/mL Recombinant Human BDNF (R&D Systems, 248-BDB, USA), 200 µM Ascorbic acid, and 100 U/mL (100 units/mL penicillin, 100 µg/mL streptomycin, and 0.25 µg/mL amphotericin B) and culture in a humidified incubator with 5% CO_2_ at 37 °C. The medium was refreshed 3 times per week. After three days in culture, the slices were infected with AAV-Ctrl and AAV-Tau. Incubate the six-well Petri dish containing the membrane insert in a humidified incubator with 5% CO_2_ at 37 °C. Replace the medium with fresh medium 3 times per week.

### 2.7. Statistical Analysis

All statistical analyses were performed using GraphPad Prism 8 (GraphPad Software, San Diego, CA, USA). Data were represented as mean ± SD. Unpaired two-tailed Student’s *t*-test was utilized for comparison of two groups. All experiments were repeated at least 3 times. *p* < 0.05 was considered statistically significant (ns: Not significant; *: *p* < 0.05; **: *p* < 0.01; ***: *p* < 0.001).

## 3. Results

### 3.1. Establishment of Monkey BSC with Neurotrophic Factors

**Figure 1:**
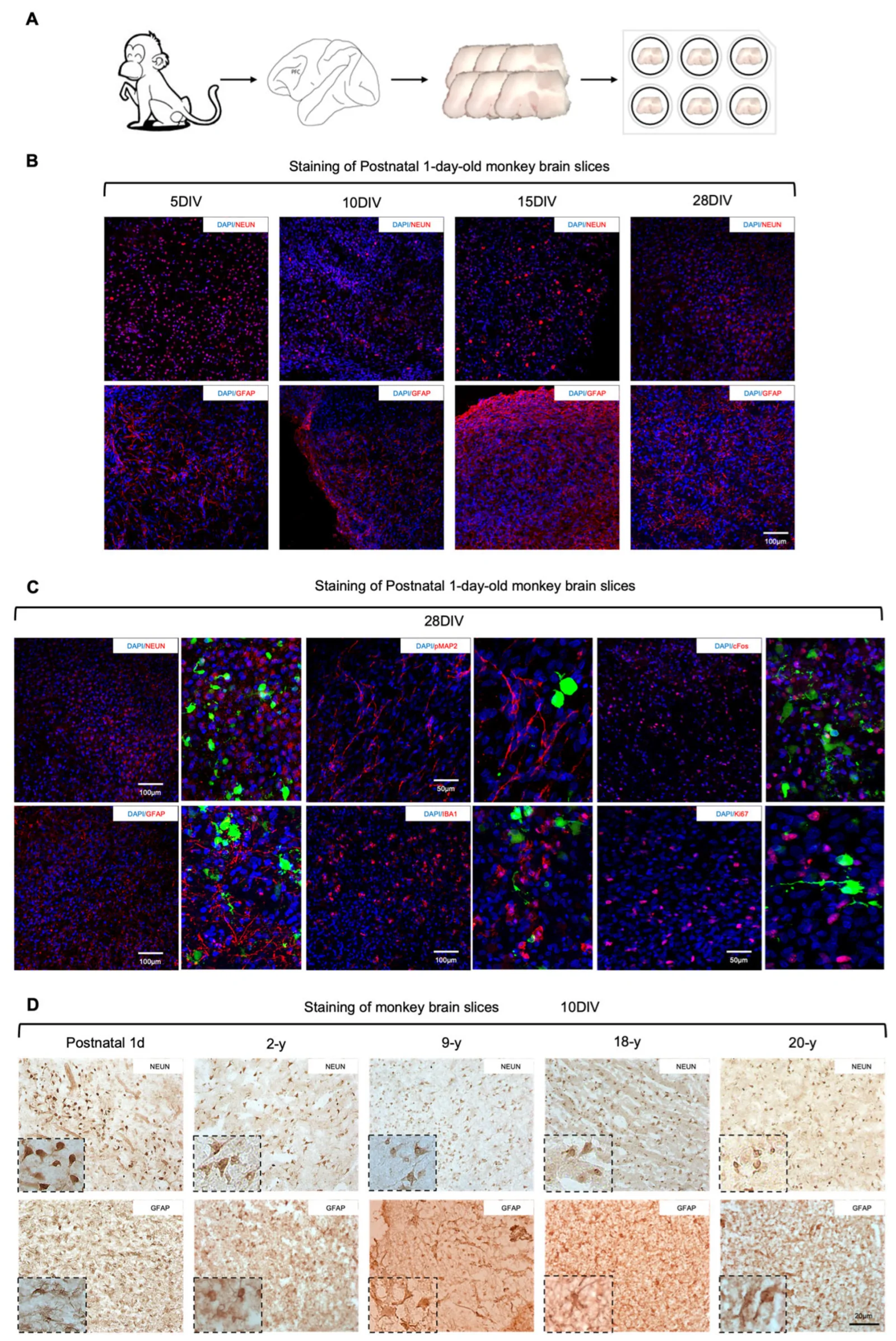
Neurotrophic factor-containing medium supports organotypic monkey brain slice culture. (**A**): Schematic description of monkey brain slice culture protocol. (**B**): Postnatal 1-day monkey brain slices harvested at 5 DIV, 10 DIV, 15 DIV, and 28 DIV; immunofluorescence staining of NeuN and GFAP was performed to detect neurons and glia respectively. (**C**): Postnatal 1-day monkey brain slices at 28 DIV, stained for neuronal markers (NeuN, pMAP2, c-Fos) and glial markers (GFAP, IBA1) in red; DAPI (4′, 6-diamidino-2-phenylindole) staining for the nucleus. (**D**): Brain slices from monkeys at different ages (postnatal 1-d, 2-y, 9-y, 18-y and 20-y) were collected at 10 DIV and immunohistochemically stained for NeuN and GFAP. Scale bar, 20 μm.

Organotypic brain slices comprise both neurons and glial cells, which may require distinct nutrient supplies. In previous studies, neurotrophic factors were routinely omitted from the culture medium for brain slice cultures (BSCs). In this study, we initially followed a standard serum-containing medium regimen without neurotrophic factors; however, after two weeks of culture, monkey cerebral tissues exhibited severe dissociation and were unsuitable for further analysis. We therefore supplemented the medium with GDNF and BDNF to better support neuronal survival. With the addition of these neurotrophic factors, both neurons and astrocytes (Figure 1A,B) were well preserved for over four weeks. MAP2 and c-Fos staining revealed fine neuronal morphology and active neuronal status at 28 days in vitro (DIV) (Figure 1B).

Given that aging is critical for neurodegeneration pathogenesis, we next applied this optimized culture medium to brain slices from monkeys at various ages, ranging from postnatal day 1 to 20 years. Both neurons and glial cells were successfully preserved for at least two weeks (Figure 1C), paving the way for investigations of age-dependent neurodegenerative diseases.

### 3.2. Optimization of AAV Serotype and Promoter for Gene Delivery

AAV is a widely used tool for gene modification in central nervous system (CNS) cells [1,2,3] and is categorized into multiple serotypes based on capsid differences, which determine their infection tropism toward specific brain regions or cell types [4,5,6]. Although several studies have investigated AAV tropism in monkeys in vivo [6,7,8,9], whether these findings are applicable to monkey brain slice cultures (BSC) remains unknown. In addition, promoter choice is critical for achieving high-level transgene expression and can influence susceptibility to gene silencing upon host cell modification.

**Figure 2:**
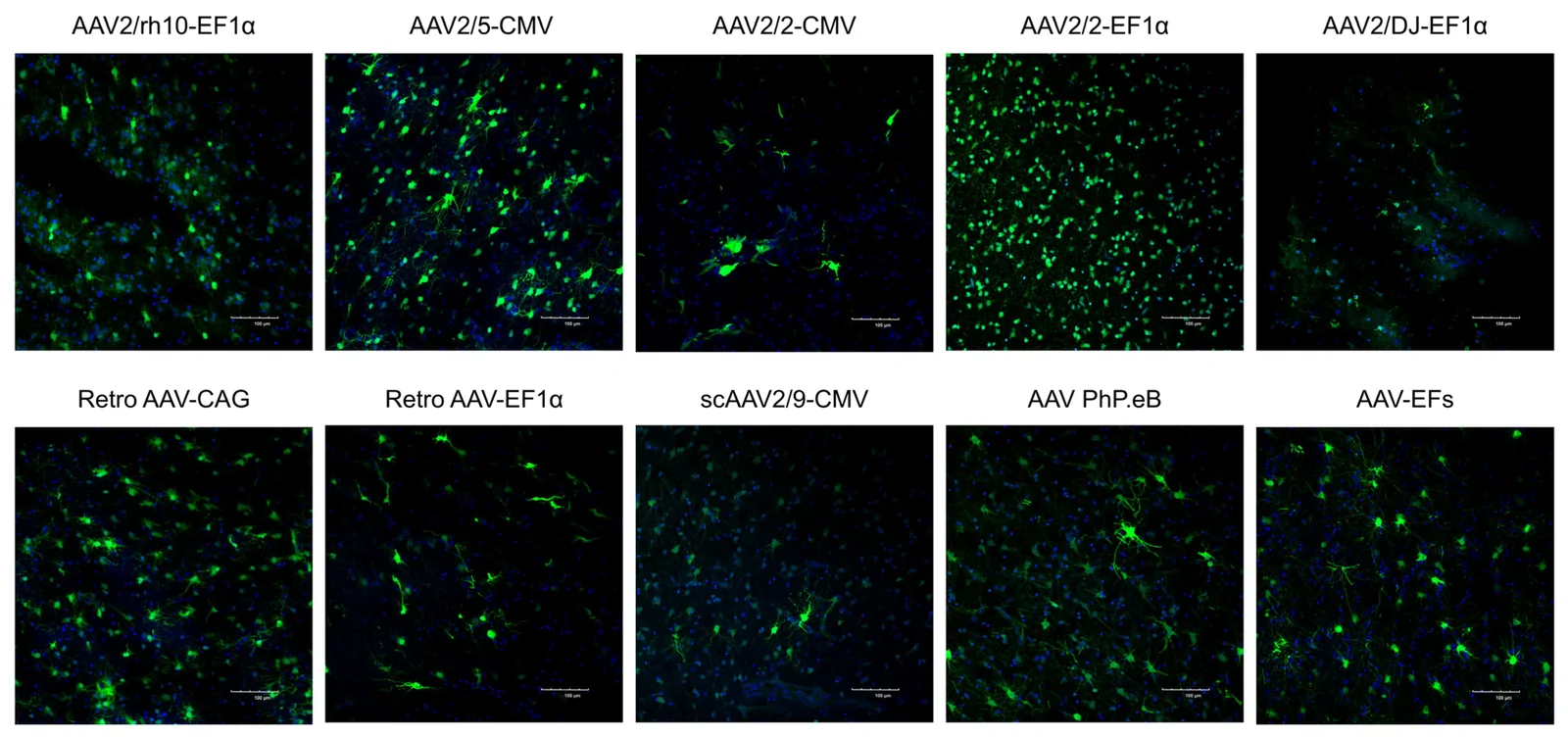
Transgene expression mediated by different AAV serotypes and promoters. AAV capsid variants 2, 5, 6, PHP.eB, rh10, and DJ with CAG, CMV, EF1α, or EFs promoters driving EGFP are applied on BSCs. Reporter GFP fluorescence is examined 10 days post-infection. Scale bar, 100 μm.

To identify the optimal AAV serotype and promoter for monkey BSC, we delivered AAV2, AAV5, AAV6, AAV9, AAVrh10, AAV PHP.eB, and AAV DJ, each driving EGFP expression under the CMV, CAG, UBC, EF1α, EFs, or CBh promoters, to BSC at a titer of 1 × 10^12^ vg/mL (Table 1). At 10 days post-infection in 9-y monkey brain slices (1 slice per virus infection, totaling 10 slices), EGFP expression was examined to identify the most effective transgenic vectors. Among the serotypes and promoters tested, AAV5 combined with the CMV promoter and retrograde AAV yielded the highest efficiency (Figure 2). All AAV vectors we tested tended to preferentially infect glial cells. We found that 7–19% of neurons and 81–93% of glial cells were infected by AAV, supporting that glial cells were preferentially infected in the cultured brain slices. In addition, we compared vector transduction profiles between tissue samples from 6 female and 3 male macaques, yet no sex-related differences were observed. This preferential infection may be attributable to the better viability of glial cells compared to neurons in brain slice culture, which allowed us to focus on glial pathology.

### 3.3. Recapitulation of Early Tau Pathology in Monkey BSC

Mounting evidence has demonstrated that neurofibrillary tangle (NFT) load and distribution are more closely correlated with the manifestation and progression of AD symptoms than amyloid-β pathology [1]. NFTs are primarily composed of hyperphosphorylated tau protein that has dissociated from microtubules. The tau protein exists as six isoforms in adult humans, but only three isoforms in mice [2], a discrepancy that may underlie the limited translational success in the AD field. Current tau transgenic mouse models fail to recapitulate prominent neuronal loss, which may lead to misleading results. Moreover, most AD postmortem specimens are obtained at end-stage disease, and fresh tissue is rarely available. Therefore, a primate model that can mirror early pathogenic events—particularly tau hyperphosphorylation (pTau)—could potentially benefit AD research and prevention strategies.

**Figure 3:**
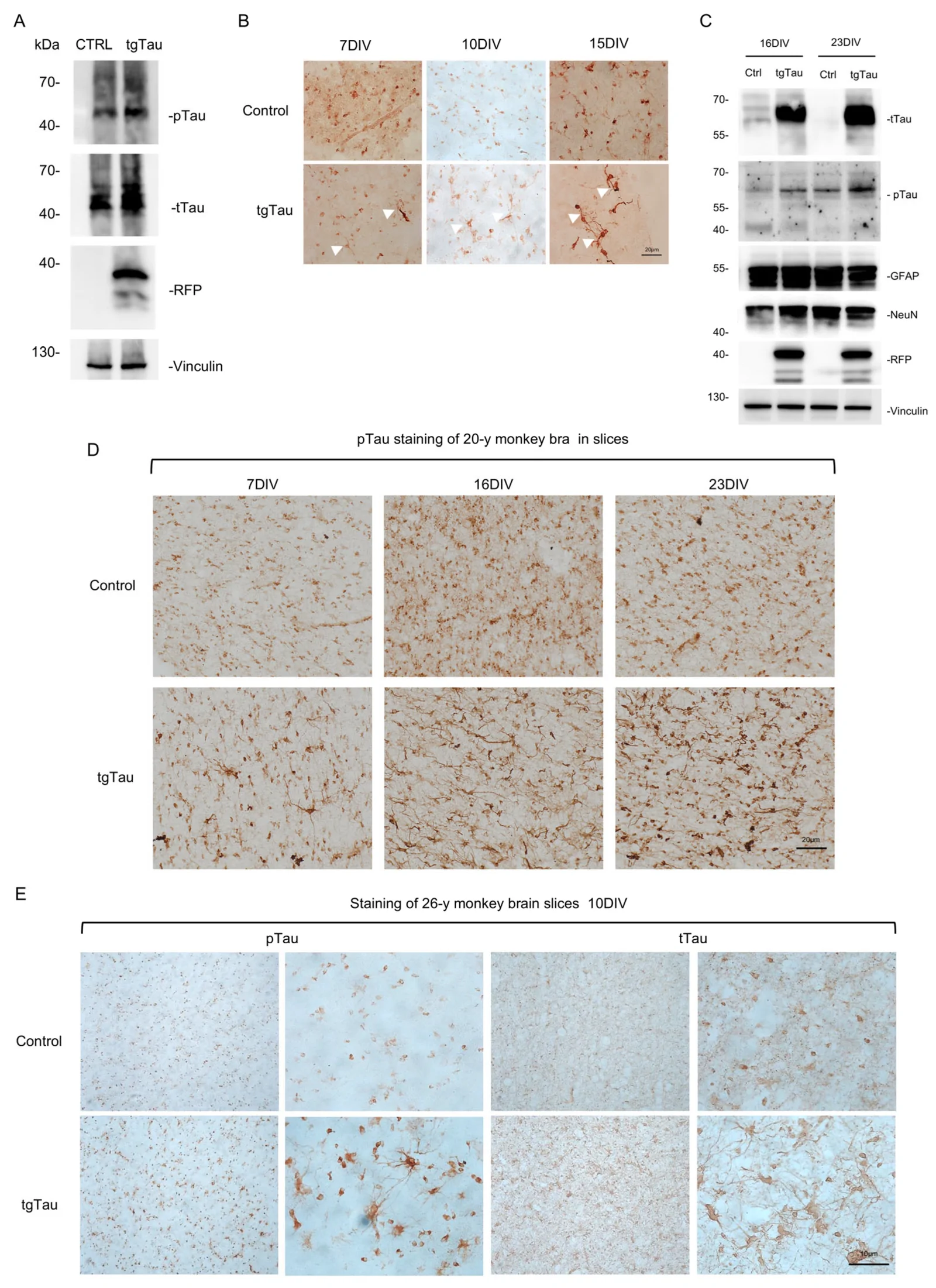

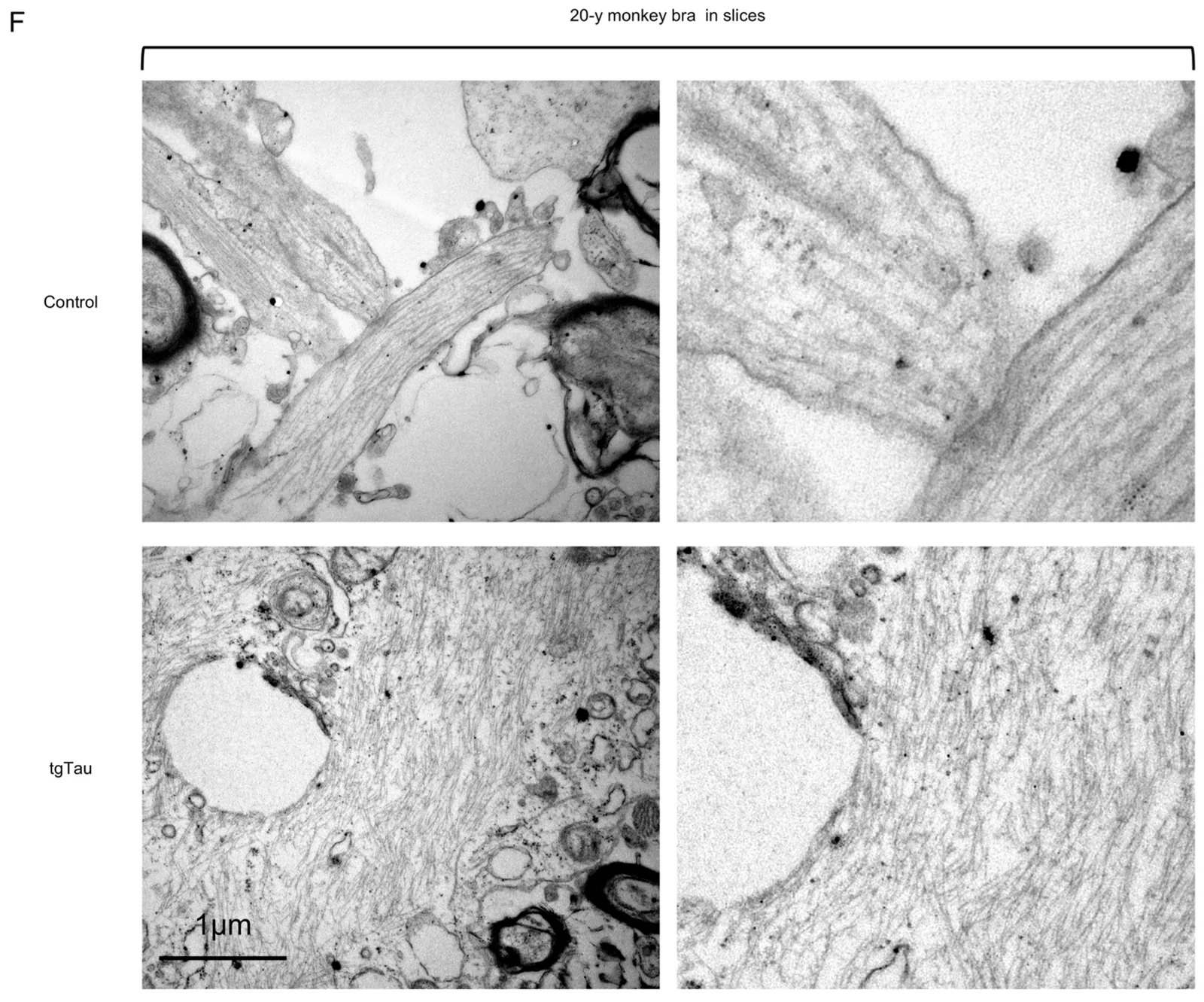
Overexpression of full-length human tau in young and old monkey BSC recapitulates the early process of neurodegeneration. (**A**): Western blot for pTau and tTau in 2-year monkey brain slices at 16DIV, wild-type control and tgTau; RFP represents transgene expression level, and vinculin serves as internal reference. (**B**): immunohistochemical staining for pTau of monkey brain sections at 7DIV,10 DIV, and 16DIV. The white triangle in the figure represents the positive signal of pTau. Scale bar, 20 μm. (**C**): Western blot of pTau and tTau in 20-year monkey brain slices at 16DIV and 23DIV. (**D**,**E**): pTau immunohistochemical staining of cultured aged monkey brain sections (7 DIV, 16 DIV and 23 DIV). (**F**): Electron microscopic examination of subcellular structure of neural filaments. Scale bar, 1 μm. The full, uncropped Western blot images are available in the Supplementary Materials.

To test whether human tau can induce classic tau pathology in monkey BSCs in an age-dependent manner, we employed AAV carrying the CMV promoter to drive overexpression (OE) of full-length human tau (2N4R) along with RFP in specimens of various ages. In young monkey BSCs at 7 days in vitro (DIV), total tau (tTau) and phosphorylated tau (pTau) were detectable by Western blot (Figure 3A). We then performed immunohistochemistry on BSCs at 7, 10, and 16 DIV. Tau phosphorylation was observed in both wild-type and overexpression samples. Transgenic tau was initially localized to the cell soma at 7 DIV, extended to axons and dendrites by 10 DIV, and 16 DIV, transgenic cells began to lose axonal and dendritic processes while the cell soma remained preserved (Figure 3B). This recapitulates the processes of tau phosphorylation and early-stage neuronal degeneration within a short time frame. When we quantified tau phosphorylation at 16 DIV and 23 DIV, we found that pTau levels increased over time, with no evident neuronal loss or astrogliosis detected (Figure 3C), suggesting that the pathology remained at an early stage. At each time point, two sheets were taken: one for Western blotting and one for immunofluorescence staining.

### 3.4. Age-Dependent Tau Pathology and White Matter Enrichment

To determine whether tau pathology is age-dependent in BSC, we also transduced tau genes into BSCs derived from aged animals (Figure 3D,E). Transgenic BSCs from aged animals (age over 18 years) showed more severe pathology than their wild-type counterparts, indicating that BSCs recapitulate age-dependent tau pathology. To examine whether microtubule structure is affected by tau pathology, we performed electron microscopy and observed abundant AD-like disorganized neural filaments (Figure 3F).

**Figure 4:**
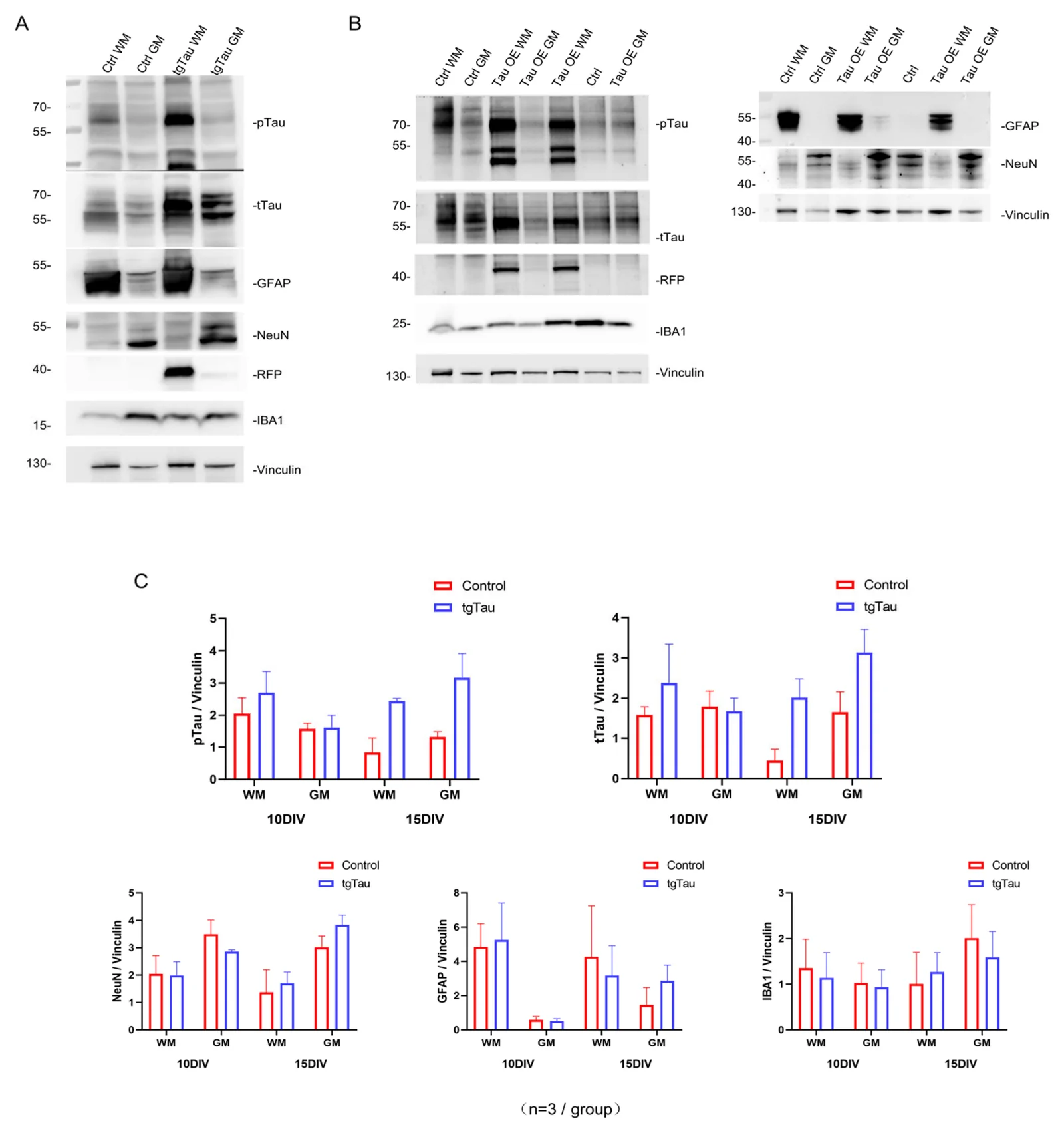
Tau pathology was enriched in white matter. (**A**): Western blot for a 26-y monkey slice with WM and GM load, respectively, at 10 DIV; the blot is probed with pTau, tau, neuronal marker NeuN and glia markers (GFAP and IBA1); RFP represents transgene expression level; vinculin serves as internal control. (**B**): Western blot of BSC at 10 DIV and 15 DIV; WM and GM were separately loaded; the blot is probed with pTau, tTau, NeuN and GFAP. (**C**) Quantification of Western blot. The full, uncropped Western blot images are available in the Supplementary Materials.

Since tau pathology is theoretically expected to initiate at proximal axonal sites, which are structurally defined as white matter (WM), we separated samples into WM and gray matter (GM) for subsequent experiments. Interestingly, we found that tau pathology was significantly enriched in WM (Figure 4A), consistent with the property that AAV preferentially infects glial cells in cultured monkey brain slices. We next compared tau pathology in WM and GM at 10 DIV and 15 DIV to assess its progression over time. During this interval, the pathology remained stable without overt progression (Figure 4B,C). At each time point, two sheets were taken to separate the cortex’s gray matter and white matter for Western blotting.

## 4. Discussion

In this study, we established an NHP brain slice culture (BSC) system and found that culturing monkey BSCs required conditions distinct from those used for rodent brain slice cultures. For rodent BSCs, high levels of horse serum are commonly used [57,63,64]; however, when we applied the same regimen to NHP BSCs, glial cells grew well but neuronal loss occurred rapidly. Previous studies have primarily focused on electrophysiology, which does not require prolonged in vitro culture. Since primary neuron cultures generally include neurotrophic factors, we supplemented our culture medium with BDNF and GDNF to adequately support both primate neurons and glia. Furthermore, we cultured brain slices from monkeys at postnatal days 1 to 28 years of age, enabling us to investigate senescence-dependent, primate-specific pathology. Although this system accommodates specimens of various ages, rapid processing is critical for successful culture; we ensured that all procedures were completed within 4 h and that specimens were maintained at 4 °C throughout.

Gene modification is essential for studying gene function, and AAV is broadly utilized to deliver genes of interest into CNS cells [61,65]. AAV serotypes are classified based on capsid residue polymorphisms. Previous studies have investigated AAV serotype tropism across different species and tissues [66,67]; however, it remains unclear which serotype and promoter combination achieves efficient gene delivery and expression in monkey BSCs. In this study, we systematically compared AAV2, AAV5, AAV6, AAV9, AAVrh10, AAV PHP.eB, and AAV DJ serotypes, each driving transgene expression under the CMV, CAG, UBC, EF1α, EFs, or CBh promoters. Among the combinations tested, AAV5 with CMV and AAV-retro with CAG were the most potent in our experimental settings.

Previous studies on brain slice cultures have predominantly used rodents, which allow multi-group in vivo experiments but differ markedly from humans in brain architecture, limiting translational validity [57,68]. Human slice cultures, in turn, rely on surgically resected lesions from epilepsy or tumor patients—specimens that are ethically constrained and often compromised by pathological microenvironments [69]. To overcome these limitations, we adopted non-human primates as experimental subjects. Primates share closer phylogenetic and anatomical homology with humans, including neuronal subtypes and neural networks, thereby providing an effective bridge between rodent basic research and human clinical pathology [8,70]. Moreover, primate brain tissue permits multi-group parallel experiments under conditions that more closely mimic human physiology, enabling systematic evaluation of interventions while making optimal use of these precious resources and avoiding biases associated with lesion tissue or rodent models.

Brain slice culture is a long-established tool in neuroscience research and has recently been applied to investigate neurodegenerative diseases in rodents. To address the issue of primate AD model scarcity, we leveraged a monkey BSC system that is amenable to gene modification to simulate AD pathogenesis in vitro. We successfully recapitulated AD-related early-stage tau pathology, paving the way for studying primate-specific mechanisms and contributing to translational research.

AD pathology typically develops several years or even decades before clinical symptoms manifest [71,72,73], making it nearly impossible to obtain clinical specimens at early pathogenic stages—a critical period for early diagnosis and the development of preventive strategies. In this study, we found that in vitro cultured monkey brain slices developed pathological tau phosphorylation within two weeks, recapitulating aspects of early tau pathology. When full-length human tau was delivered into monkey BSCs, tau pathology was significantly enhanced. Furthermore, we observed that tau hyperphosphorylation-mediated pathology was primarily enriched in white matter, where axons are densely concentrated. We also detected axonal loss preceding somatic degeneration over time in BSCs from the same animals, indicating an early pathological stage. Given the predominant glial tropism of AAV vectors observed in this model, we cannot exclude the possibility that the prominent white matter tauopathy is due to higher AAV transduction efficiency in axonal compartments or glial cells. More likely, this regional tau enrichment may represent a synergistic outcome of pathological alterations occurring in both neurons and glial cells. Collectively, monkey BSCs may serve as a valuable platform for studying early pathogenesis and for screening candidate therapeutic drugs.

**Figure 5:**
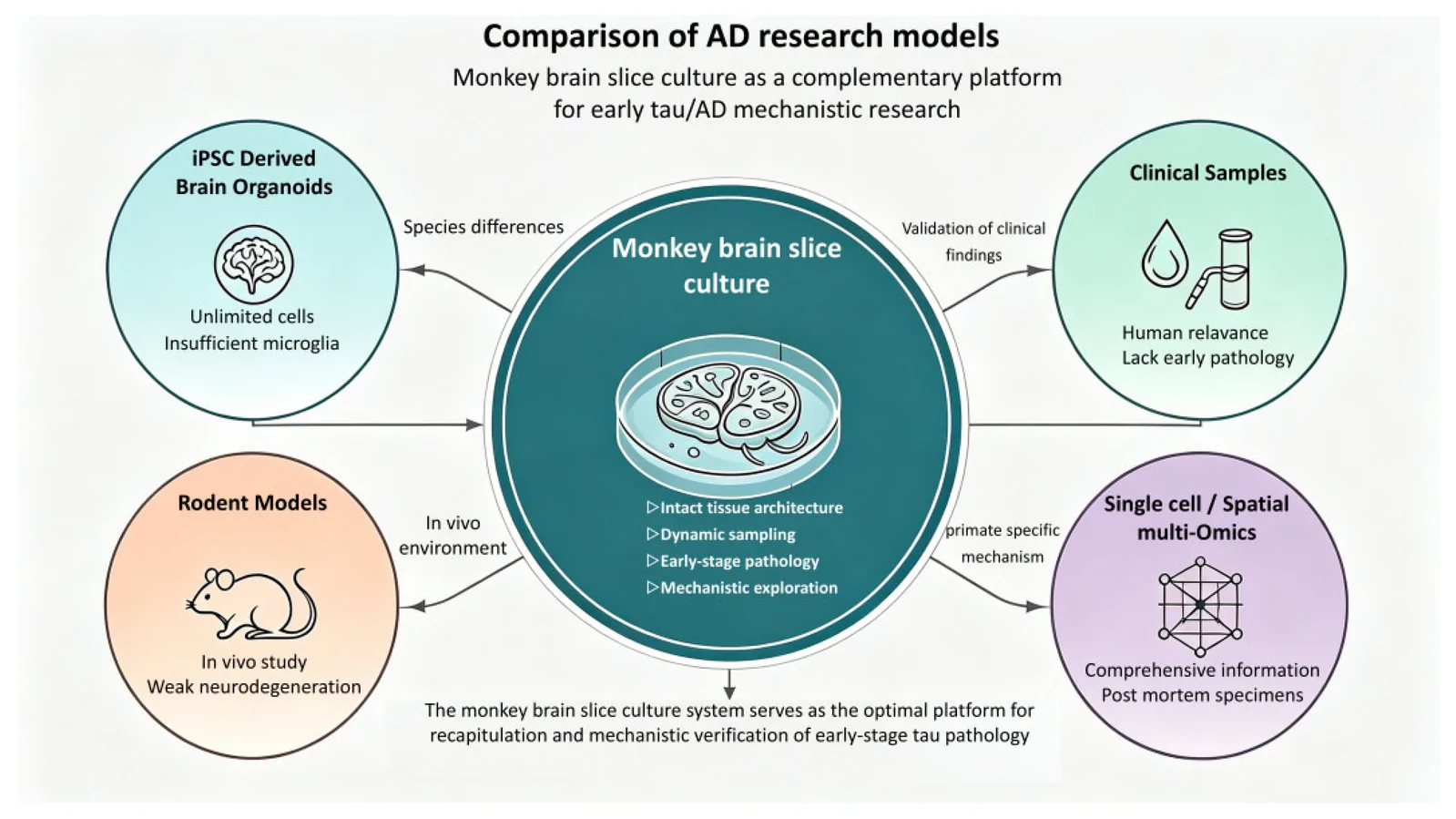
Schematic comparison and illustration of monkey brain slice culture model with other models.

Recently, multiple sophisticated technologies have greatly advanced AD investigations, including patient-induced pluripotent stem cell (iPSC)-derived brain organoids [74], newly validated prodromal AD biomarkers [75], high-sensitivity neuroimaging probes [76], and single-cell or spatial multi-omics approaches [77,78]. iPSCs provide virtually unlimited cellular resources for longitudinal studies, while brain organoids recapitulate the major cellular components of cerebral tissue and facilitate the exploration of synergistic interactions between neurons and glial cells. However, most current brain organoid models fail to incorporate microglia and oligodendrocytes, which critically contribute to AD pathogenesis. Although fluid biomarker screening and neuroimaging techniques enable early AD diagnosis and preventive evaluation, their clinical validation and mechanistic exploration remain challenging. In this context, the monkey brain slice platform serves as an alternative experimental system to validate these clinical findings and dissect the underlying molecular mechanisms. Similarly, single-cell and spatial multi-omics analyses offer comprehensive molecular profiles of AD but are largely limited to postmortem specimens, precluding the capture of dynamic neurodegenerative processes. By contrast, the monkey brain slice model allows for longitudinal sample collection and enables the characterization of the cascade of early pathological progression. Furthermore, with the rapid advancement of artificial intelligence (AI), integrative AI-based analysis of multi-dimensional data from diverse experimental models, such as traditional rodent models, iPS-derived organoids, and clinical data (Figure 5), is poised to revolutionize AD research and substantially elevate the depth and translational value of this field [79].

Although the NHP BSC system is a facile and efficient platform for investigating tau pathology, several limitations should be addressed in future studies to advance our understanding of AD. First, longer culture durations are needed to observe late-stage pathological changes. Second, more efficient strategies for transgene expression in neurons need to be developed. Third, faster sectioning and processing methods are required, as current low-speed vibration sectioning limits both the number and yield of BSCs obtainable from each animal. Fourth, cryopreservation techniques should be developed for long-term storage of BSCs. In addition, it is important to bear in mind that this system operates without blood flow, which in vivo would facilitate the recruitment of peripheral immune cells such as T cells to the brain and contribute to pathogenesis. Also, regional tissue culture loses brain region communication so it is impossible to study how pathological tau seeding occurs across functional networks. Furthermore, the sectioning procedure itself introduces acute injury to the slices, which may confound data interpretation. Therefore, these factors should be carefully considered when extrapolating findings from the BSC system.

## 5. Conclusions

In summary, the present study established a culture system capable of sustaining viable nonhuman primate brain slices for up to four weeks. Using this ex vivo platform, we systematically profiled AAV tropism across distinct serotypes and promoters and confirmed a preferential transduction of glial cells in cultured monkey brain tissues. Notably, exogenous expression of mutant human P301L tau in monkey brain slices successfully recapitulated the key pathological characteristics of early tauopathy, including tau hyperphosphorylation, aberrant tau accumulation, and disrupted microtubule architecture. Collectively, this primate brain slice model bridges a critical translational gap between traditional rodent models and human tau research. It provides a unique and tractable platform to uncover primate-specific molecular mechanisms underlying early tau pathogenesis and offers valuable insights to facilitate future translational investigations and therapeutic development for neurodegenerative diseases.

## Figures and Tables

**Table 1:** Comparison of serotypes and promoters in monkey brain slice culture.

Serotype	Promoter	Effect
AAV9	CMV	++
AAV9	EFs	++
AAV2/rh10	EF1α	+
AAV2/DJ	EF1α	++
AAV2/5	CMV	+++
AAV2/2	CMV	++
AAV2/2	EF1α	++
AAV2/6	CBh	+
AAV retro	CAG	+++
AAV retro	EF1α	++

+ low, ++ moderate, +++ high transduction efficiency.

## Data Availability

The original contributions presented in this study are included in the article/Supplementary Materials. Further inquiries can be directed to the corresponding authors.

## Supplementary Materials

The following supporting information can be downloaded at: https://www.mdpi.com/article/10.3390/biology15171496/s1, Supplementary File: Full Western blot images.
